# Method of Levodopa Response Calculation Determines Strength of Association With Clinical Factors in Parkinson Disease

**DOI:** 10.3389/fneur.2018.00260

**Published:** 2018-05-17

**Authors:** Marcus Pieterman, Scott Adams, Mandar Jog

**Affiliations:** ^1^Department of Clinical Neurological Sciences, Schulich School of Medicine & Dentistry, University of Western Ontario, Canada; ^2^School of Communication Sciences and Disorders, University of Western Ontario, Canada

**Keywords:** Parkinson disease, levodopa, levodopa response, levodopa challenge test, Unified Parkinson Disease Rating Scale, disease duration, age

## Abstract

**Background:**

The levodopa challenge test is routinely used in Parkinson disease (PD) to determine a patient’s motor improvement following levodopa administration [levodopa response (LR)]. LR is most commonly reported as a percent OFF to ON change in the Unified Parkinson Disease Rating Scale (UPDRS) part III score, and occasionally as an absolute difference in score. This inconsistency in LR determination alters how clinical factors such as patient age and disease duration are understood in relation to LR in PD.

**Objective:**

The aim of this study was to compare the calculation of the LR as either a percent change or difference in UPDRS-III motor score between OFF and ON medication. These two scores were then used to correlate to disease duration, patient age, levodopa duration, levodopa equivalent dose (LED), OFF score, cognition, mood, gait, and quality of life (QOL).

**Methods:**

70 PD patients underwent the levodopa challenge test. The UPDRS-III motor examination was performed in the defined OFF and ON medication states to determine LR. Each patient was assessed after 12–14 h without anti-parkinsonian medication and then given three 100/25 mg levodopa/carbidopa tablets. LR was reported as both a difference in score [OFF − ON; absolute LR (aLR)] and as a percent change in score [(OFF − ON)/OFF*100%; %LR]. Patients completed the following non-motor symptom assessment scales: Montreal Cognitive Assessment, Freezing of Gait Questionnaire, Activities-specific Balance Confidence Scale, Parkinson’s Disease Questionnaire, and Geriatric Depression Scale. The effect of the LR calculation method was correlated to the clinical measures.

**Results:**

The aLR was significantly associated with disease duration (*r* = 0.40), levodopa duration (*r* = 0.47), OFF motor score (*r* = 0.58), and LED (*r* = 0.31), but not age. The aLR was also found to have a significant relationship with clinical scales assessing cognition (*r* = 0.41), freezing of gait (*r* = 0.35), QOL (*r* = 0.40), and depression (*r* = 0.30). By contrast, the more commonly used %LR demonstrated no significant relationships with any of the variables tested.

**Conclusion:**

Although the %LR is more commonly employed in clinical protocols and research studies, the aLR is the superior method for reporting motor response to levodopa in PD given its significant associations with the clinical factors evaluated.

## Introduction

Parkinson disease (PD) is the second most common neurodegenerative disorder after Alzheimer’s disease (1, 2). The disease is accompanied by an array of disabling motor and non-motor symptoms. Fortunately, many suffering from PD receive significant motor benefit from levodopa, the gold-standard treatment for Parkinson’s disease. Year 2017 marks levodopa’s 50th anniversary since its therapeutic value in managing PD motor symptoms was first demonstrated (3, 4). In both clinical and research settings, the extent of motor benefit provided by a single-dose of levodopa is often sought using the levodopa challenge test. The motor improvement observed as a change in part III of the Unified Parkinson Disease Rating Scale (UPDRS) during this test is commonly referred to as the “levodopa response” (LR). The reduction in the UPDRS part III score from OFF to ON is thought to indicate how well a patient is responding to levodopa at any point along their disease course.

The calculation of LR remains inconsistent in the literature. Regardless of the scale used to quantify a PD individual’s motor severity, the LR is sometimes reported as a difference between OFF and ON scores [absolute LR (aLR)] and in other instances, it is calculated as a percent change from OFF to ON (%LR). In studies correlating LR with variables such as patient age and disease duration, Clissold et al. (5) and Ganga et al. (6) reported the aLR whereas Durso et al. (7) and Aygun et al. (8) used the %LR. According to the core assessment program for surgical interventional therapies in Parkinson’s disease (CAPSIT-PD protocol), the %LR is used. This protocol recommends individuals demonstrate a minimum 33%LR as part of the screening process for determining deep brain stimulation (DBS) candidacy (9). However, there is no clear consensus as to which method of LR calculation (aLR or %LR) provides the most clinically relevant information. Highlighting the more sensitive calculation method may assist physicians in making more highly informed treatment decisions and guide researchers in selecting optimal reporting methods to elucidate underlying relationships.

Clinicians turn to factors such as age and disease duration in guiding their decisions to adjust current treatments and initiate more invasive procedures like DBS (9, 10). Reference to an LR calculation method more highly associated with these patient characteristics would serve to enhance clinical accuracy and judgment in making such decisions. Providing a construct of how the LR changes with respect to age, disease duration and similar factors would certainly be of therapeutic value; however, this may depend on the way in which the LR is reported. A 20-year longitudinal study by Clissold et al. (5) reports that OFF and ON motor scores rise in parallel during early years of disease. However, after at least 3 years of treatment, they found that the amplitude of motor response (aLR) widens due to increasing severity of OFF medication motor scores. They concluded that as disease duration increases, PD patients do not lose their capacity to respond to levodopa. A follow-up report by Ganga et al. (6) on this longitudinal study corroborated the finding that the aLR is maintained with increasing disease duration. Furthermore, they found that the aLR was significantly larger in amplitude after 15 years of PD as compared with those of 5 years of disease or less. Neither Clissold et al. (5) nor Ganga et al. (6) made any mention of the relationship between age and aLR. By contrast, studies by Durso et al. (7) and Aygun et al. (8) employed the %LR and found that age negatively correlated with %LR and that disease duration was not significantly associated. Hence, these discrepancies may be attributed to the way in which the LR was calculated between studies.

The aim of this cross-sectional study was to compare the two methods of absolute versus % change as a measure of LR. The two methods were also compared on the ability to be affected by age and disease duration, important clinical characteristics for managing treatment. In addition, the association between LR and levodopa exposure duration, OFF motor scores, daily levodopa equivalent dose (LED), and various clinical scales was investigated.

## Materials and Methods

### Study Participants

Seventy PD participants were recruited from the Movement Disorders Centre, University Hospital, London, ON, Canada. This study was approved by the Human Research Ethics Board (REB #107253) of Western University. Participants were included based on the following criteria: (1) have been diagnosed with idiopathic PD for at least ≥2 years; (2) be 45–85 years of age; (3) have been on stable doses of anti-Parkinson medication, including any levodopa preparation (stable doses indicate that no adjustments to medications have been made within the last 6 months); and (4) able to give informed consent. Participants were excluded on the following criteria: (1) history of any surgical intervention for treating PD (i.e., DBS, Duodopa pump); (2) extreme physical disability that impairs mobility assessment; (3) history or current diagnosis of a psychiatric condition requiring hospitalization; (4) pregnant, planning on becoming pregnant, or breastfeeding; and (5) deemed unable to understand or speak sufficient English.

### Levodopa Challenge Test

Participants underwent the levodopa challenge test according to the CAPSIT-PD protocol (9). This test involved participants visiting the research center after 12–14 h without anti-parkinsonian drugs (practically defined “OFF” state). Participants were instructed to take their last dose of anti-parkinsonian medication at 8:00 p.m. on the night before the study and arrive at 9:00 a.m. the following morning. Dopamine agonists were off for 24 h. This was to allow for an appropriate washout of levodopa.

Upon arrival, a detailed medical history of the participants’ Parkinson’s disease was completed. This involved confirming the patient’s age, gender, date of PD diagnosis, date of first intervention with levodopa, and current medications. Current medications were recorded as the daily LED which uses conversion factors provided by Tomlinson et al. (11). Next, the motor examination portion (part III) of the Movement Disorders Society UPDRS was performed to provide a clinically defined-OFF motor score. After the motor examination was completed, participants were instructed to take three 100/25 mg levodopa/carbidopa tablets. Participants were then reassessed using the UPDRS-III when found to be in their clinically defined-ON medication state (when both the patient and clinical rater agree that the individual is receiving the highest level of therapeutic benefit from the administered levodopa or approximately 45–60 min after levodopa is given). Hyperkinesia was not assessed. This was the ON state motor score. The LR was then calculated as both a difference in score (OFF − ON; aLR) and as a percent change in score [(OFF − ON)/OFF*100%; %LR].

### Clinical Questionnaires

A series of clinical questionnaires that assessed several non-motor features were completed following the final motor assessment in the ON state. All clinical questionnaires used can be found in Table 1, which include Montreal Cognitive Assessment (MoCA), Freezing of Gait Questionnaire (FOG-Q), Activities-specific Balance Confidence Scale (ABC), Parkinson’s Disease Questionnaire (PDQ-8), and Geriatric Depression Scale (GDS).

**Table 1:** Clinical measures of the 70 PD participants enrolled.

Clinical measure	Mean ± SD	Range
Age	66.13 ± 7.2 years	47–82 years
Sex; female: male	19 F: 51 M	–
Disease duration	9.16 ± 4.3 years	2–18 years
Levodopa duration	7.49 ± 4.2 years	1–17 years
LED	988.42 ± 437 mg	300–2,200 mg
OFF; MDS UPDRS-III Motor Score	30.64 ± 10.23	6–60
ON; MDS UPDRS-III Motor Score	16.57 ± 8.17	3–49
aLR; OFF − ON	14.07 ± 6.07	3–29
%LR (OFF − ON)/OFF*100 (%)	46.80 ± 15.03%	18.33–88.88%
MoCA	25.37 ± 3.53	14–30
FOG-Q	7.47 ± 5.10	0–20
ABC	77.29 ± 18.85%	30–100%
PDQ-8	30.58 ± 16.84	3.13–62.5
GDS	10.19 ± 6.89	0–26

*SD, standard deviation of the mean; LED, levodopa equivalent dose; MoCA, Montreal Cognitive Assessment; FOG-Q, Freezing of Gait Questionnaire; ABC, Activities-specific Balance Confidence Scale; PDQ-8, Parkinson’s Disease Questionnaire; GDS, Geriatric Depression Scale; UPDRS, Unified Parkinson Disease Rating Scale; LR, levodopa response; aLR, absolute LR*.

*Levodopa duration refers to time since first intervention with levodopa*.

*OFF refers to the state in which a participant has been without anti-parkinsonian medication for at least 12 h*.

*ON refers the state in which both the participant and clinical rater agree that the participant is receiving the highest level of therapeutic benefit from the administered levodopa or approximately 45–60 min after levodopa is given*.

### Analyses

**Figure 1:**
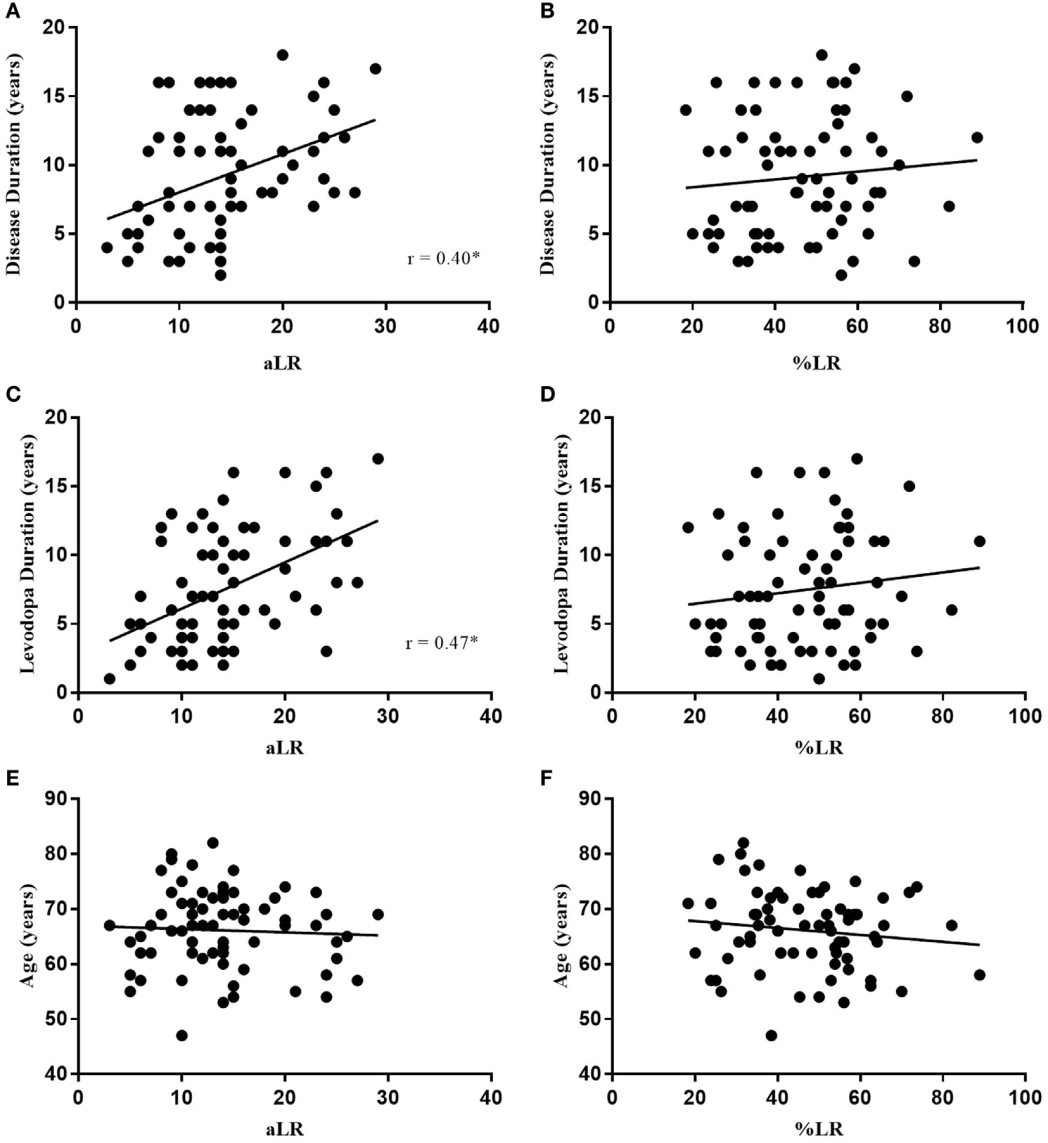
Absolute LR (aLR) is significantly associated with disease duration **(A)** and levodopa duration **(C)** where the %LR is not **(B,D)**. Neither is correlated with age **(E,F)**. Spearman’s correlation coefficient is represented as “r” on plots **(A–F)**. Black line indicates line of best fit.

**Figure 2:**
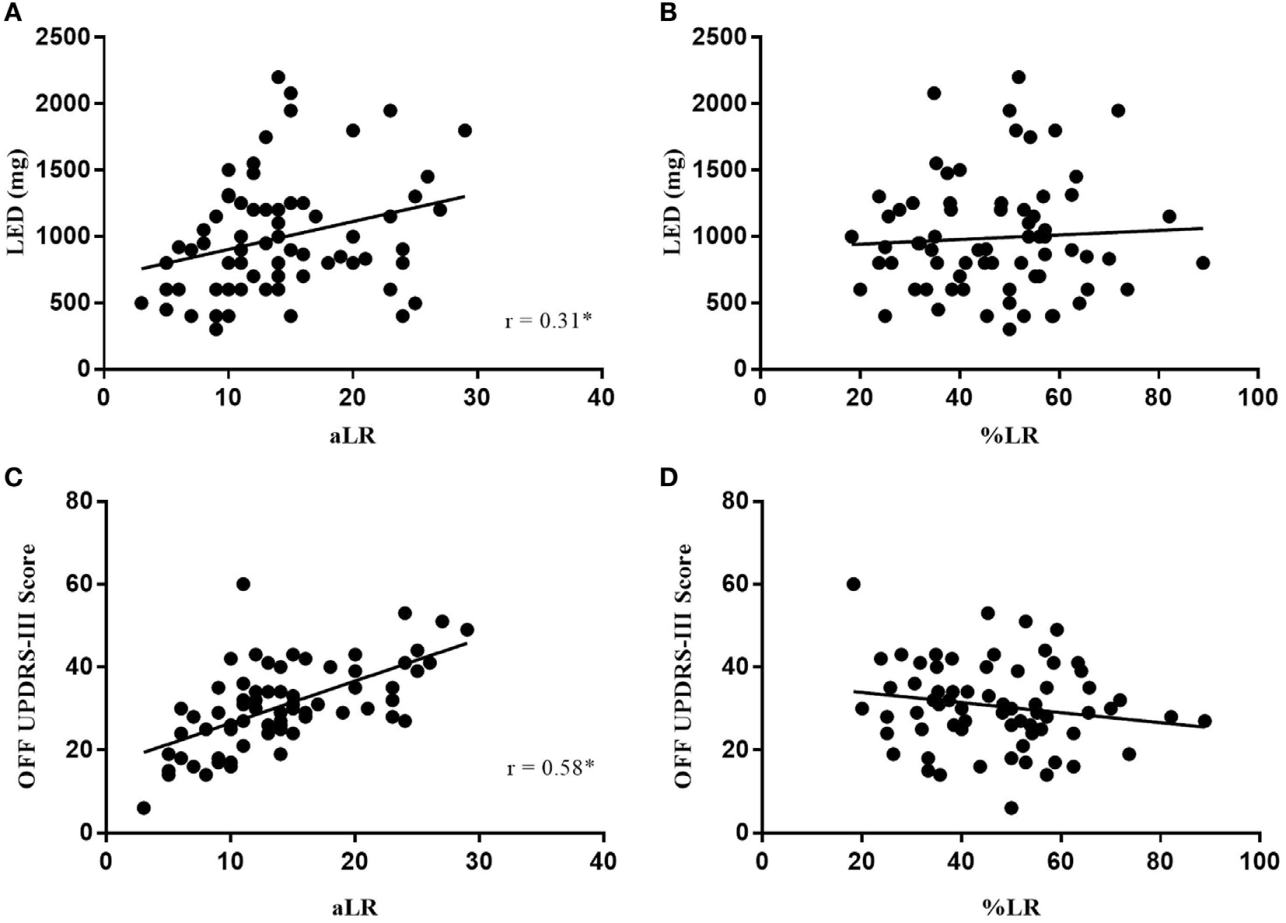
Absolute LR (aLR) is significantly associated with LED **(A)** and OFF motor scores **(C)** where %LR is not **(B,D)**. Spearman’s correlation coefficient is represented as “r” on plots **(A–D)**. Black line indicates line of best fit.

**Figure 3:**
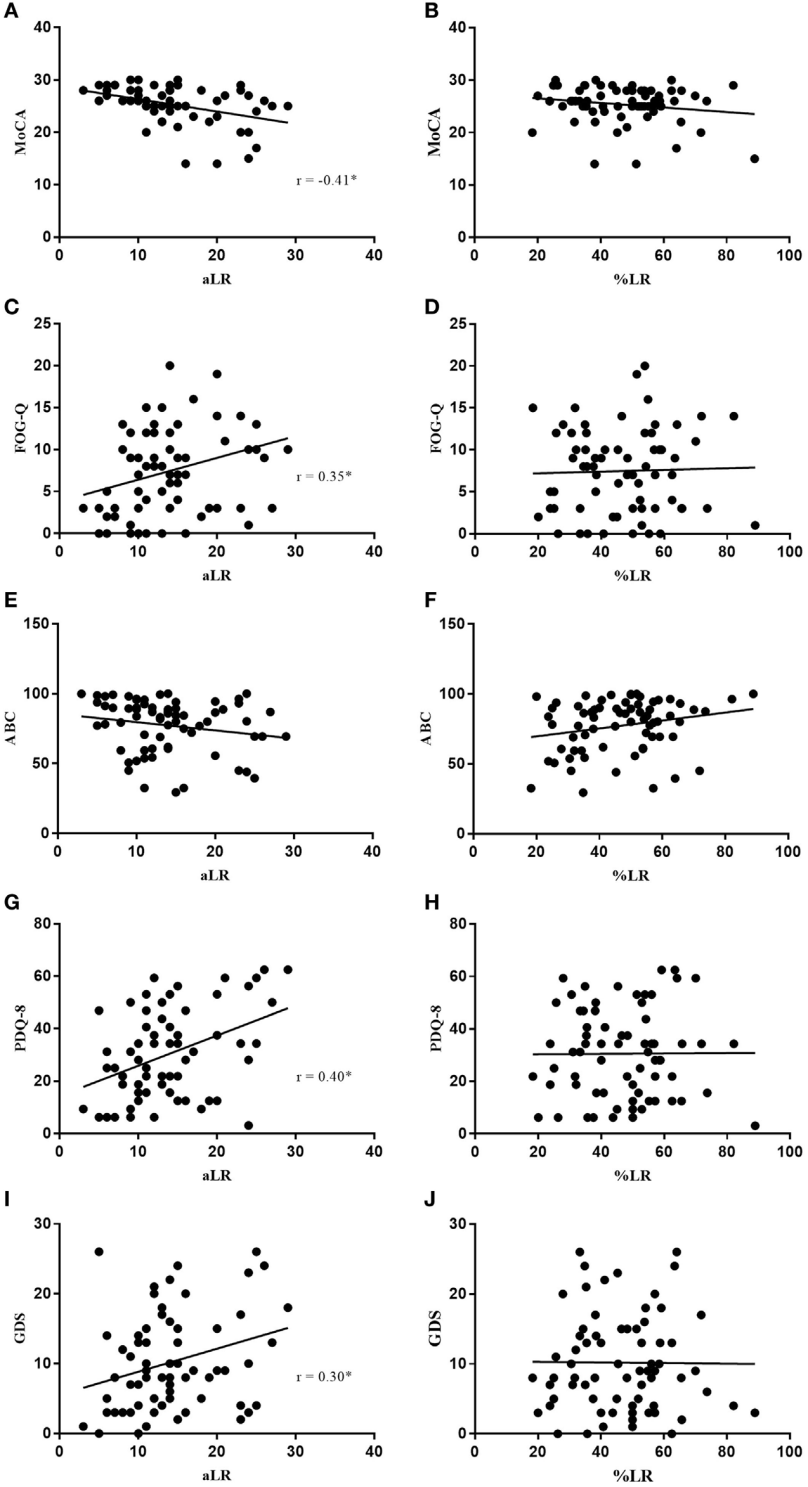
Absolute LR (aLR) is significantly associated with MoCA **(A)**, FOG-Q **(C)**, PDQ-8 **(G)**, and GDS **(I)** where %LR is not **(B,D,H,J)**. Neither aLR, nor %LR are associated with ABC scale **(E,F)**. Spearman’s correlation coefficient is represented as “r” on plots **(A–J)**. Black line indicates line of best fit.

GraphPad Prism 7.00 was used for all statistical analyses performed. As the data were not found to be normally distributed, Spearman’s rank-order correlation was used in Figures 1–3 rather than Pearson’s correlation coefficient. The significance threshold was set at *p* < 0.05 for all statistical analyses performed.

## Results

The 70 PD participants in the analysis included 19 females and 51 males. Clinical outcomes for all participants can be found in Table 1.

In Figure 1, the LR measured as a percent change (%LR) in OFF to ON UPDRS-III scores did not significantly correlate with disease duration (*p* = 0.291), levodopa duration (*p* = 0.245), or age (*p* = 0.325). Similarly, %LR did not significantly correlate with LED (*p* = 0.1779) or OFF UPDRS-III scores (*p* = 0.8515) in Figure 2. In summary, the %LR was not significantly associated with any of the variables tested in Figures 1 and 2.

However, the LR measured as a difference in score from OFF to ON (aLR) in Figures 1 and 2 had a significant positive correlation with disease duration (*r* = 0.40, *p* = 0.0005), levodopa duration (*r* = 0.47, *p* < 0.0001), LED (*r* = 0.31, *p* = 0.0097), and OFF UPDRS-III scores (*r* = 0.58, *p* < 0.0001). Among the variables tested against aLR, age was the only one found not to have a statistically significant correlation. According to the lines of best fit, the aLR increases by 3.6 UPDRS-III points per year after PD diagnosis and by 2.97 UPDRS-III points per year after initial intervention with levodopa. Furthermore, LED increases by 21 mg for every 1-point increase in aLR and LED increases by 100 mg each year since first intervention with levodopa. Finally, the aLR increases in a near 1:1 ratio with OFF medication UPDRS-III total scores.

In Figure 3, increasing aLR was significantly associated with a decline in cognition (MoCA; *r* = −0.41, *p* = 0.0005) and perceived quality of life (QOL) (PDQ-8; *r* = 0.40, *p* = 0.0007), and an increase in both freezing of gait (FOG-Q; *r* = 0.35, *p* = 0.0031) and depression (GDS; *r* = 0.30, *p* = 0.0113). Once again, %LR did not significantly correlate with any of these clinical questionnaires used. Patient confidence in completing daily activities of living (ABC) was neither associated with aLR nor %LR.

## Discussion

The extent of responsivity of motor symptoms to levodopa is crucial in management of PD. It is expected that this LR would decline with duration of disease and it is this decline that is an important indicator suggestive of disease progression. The levodopa challenge test was incorporated into the CAPSIT-PD protocol in 1999 and used to screen PD patients for DBS, relying on the %LR (9). Despite the widespread use of the LR, little attention has been paid as to how it is calculated. Whether the absolute change (aLR) or percentage change (%LR) in part III of the UPDRS is a better measure remains unsupported. A more reliable measure of LR which better correlates with clinically relevant variables such as disease duration and non-motor symptoms would allow for a more accurate determination of the disease state. This study was undertaken to test these two methods of LR calculation and determine which was more strongly associated with important disease characteristics.

The acute motor response to levodopa following a minimum 12-h washout period reported as a percent change (%LR) in motor scores from OFF to ON medication is the most commonly used measure of LR. However, calculating LR as a percentage faces the arithmetical issue of having an identical percentage decrement with the possibility of a large difference in the actual quantity of change. Therefore, a patient early in disease (patient A) with a UPDRS-III OFF score of 15 and an ON score of 10 has a 33.33%LR, but only a 5-point aLR. By contrast, a patient with more advanced PD (patient B) who improves from a score of 30 OFF medication to 20 ON medication also has a 33.33%LR, but their aLR is twice that of the first patient’s. According to the CAPSIT-PD protocol, patient A and B have the same response—33.33%. Whereas if the aLR is used, patient B had the better response to medication (a 10-point UPDRS-III difference versus 5). Thus, reporting the %LR alone may be misleading.

This simple difference in calculation might explain why Clissold et al. (5) and Ganga et al. (6), who both employed the aLR, found a significant relationship between LR and disease duration, whereas Durso et al. (7) and Aygun et al. (8) used the %LR and found no relationship with disease duration. In this study, the %LR was not found to have any significant association with disease duration, levodopa duration, age, LED, OFF scores, or any of the clinical questionnaires (Figures 1–3). In comparison, aLR significantly correlated with all factors except age and the ABC scale. Hence, aLR appears to better represent aspects of PD than %LR and may serve as a more sensitive means of reporting the LR.

This study shows that the %LR neither correlated with age nor disease duration. Aygun et al. (8) found that %LR did not correlate with disease duration; however, they did find a weak negative correlation with age. The %LR’s correlation with age in their study may have been the result of their experimental design. Aygun and colleagues acknowledge that their PD cohort consisted mainly of individuals of 5–15 years of disease duration and that the retrospective methodology naturally resulted in a biased sample population. Their study retrospectively reviewed the %LR of 54 candidates who were screened for STN-DBS, skewing the representation of the general PD population to those who fit basic criteria for DBS surgery. Although only 37 of 54 patients in their study went forward with surgery, the cohort likely does not accurately reflect the general PD population. Furthermore, only two patients were included with less than 5 years of PD. In our study, 11 patients with less than 5 years of PD were included and patients were randomly selected in efforts to represent the general PD population. Durso et al. (7) also found a negative correlation between age and the %LR in a sample size of 47. To the best of our knowledge, there have only been three studies (including this work) investigating age as it relates to the %LR. Future studies are warranted to settle the effects of age on the %LR.

Concerning the aLR, the present results demonstrated that change in the motor UPDRS-III score (amplitude of motor response) increases with increasing disease duration (Figure 1A). This substantiates results found in the 20-year longitudinal study reported on by both Clissold et al. (5) and Ganga et al. (6) as they also concluded aLR increases with disease duration. Our results further support their conclusion that a significant response to levodopa is still seen late in disease. However, it should be noted that late-stage PD is often associated with significant levodopa-induced motor complications such as motor fluctuations and dyskinesia which were not assessed in this study (12). Therefore, levodopa therapy in late-stage PD may be of limited treatment utility for some, at which point interventions such as DBS may be indicated.

Several mechanisms may be responsible for the maintenance in amplitude of response as degeneration occurs. One mechanism might include the upregulation of postsynaptic striatal D2 receptors as discussed by Aygun et al. (8). In multiple system atrophy, especially MSA-P, both the striatum and substantia nigra pars compacta (SNc) degenerate (13). Imaging has shown significant loss of pre- and postsynaptic striatal D2 receptor binding in MSA (14, 15). Given the striatal degeneration seen in MSA-P, it is well established that these patients have a poor response to levodopa. By contrast, the striatum of PD patients does not face the same degree of degeneration (16) and positron emission tomography studies have shown an upregulation of striatal D2 receptors (14). Unlike in MSA-P, the intact striatum of PD patients may in part be responsible for maintaining a good aLR as nigral terminals degenerate or disease duration increases.

A higher aLR was also shown to be associated with worsening cognition, QOL, depression, freezing of gait, and an increase in LED (Figure 3). This aligns with the finding that aLR most strongly correlated with OFF motor scores. It suggests that patients exhibiting higher amplitudes of LR have a more severe baseline motor score and as discussed by Lees (17), OFF scores are a good representation of SNc degeneration. Patients would then require an increase in LED to manage their worsening motor symptoms. Therefore, patients with higher aLR’s have likely progressed further in disease, increasing the likelihood of Lewy body pathology being present in other critical areas. According to Braak staging, amygdala and cortical involvement account for the associated behavioral changes often seen in later stages of PD (18). Hence, a high aLR may indicate progression to a more advanced disease state, explaining the observed decline in cognition, mood, gait, and rise in LED. Moreover, PD is increasingly being considered as a multisystem condition whereby neurotransmitter systems beyond the dopaminergic system may be responsible for the presentation of non-motor symptoms (19). Degeneration of serotonergic and adrenergic systems could in part account for the observed increase in depression (20), and deterioration of the cholinergic system may explain the decline in cognition (21). Finally, patients with decreased cognitive functioning, severe motor disability, and higher levels of depression may naturally be more inclined to report a reduced QOL. This would explain the correlation between the observed decline in perceived QOL and increasing aLR.

Although 70 participants were included in this study, a higher *n*-value may be needed to provide a better representation of the general Parkinson’s population. A higher *n*-value would also help to confirm the conclusive inference that the aLR is a stronger reporting method than the %LR. Furthermore, 73% of the included participants were male, providing a disproportionate representation of the male sex. Therefore, our results may provide more insight into the LR of males than females. However, it is unclear at this time if disease mechanisms differ substantially between genders (22).

### Conclusion

This study results demonstrate that although %LR is employed by the CAPSIT-PD protocol and is commonly used in research studies, aLR is the superior calculation method for reporting motor improvement in the levodopa challenge test for Parkinson’s disease. Remarkably, the %LR was not found to be associated with any of the variables used. Therefore, clinical and research measures of LR should consider calculating both the %LR and the aLR, with more emphasis on the aLR. Dual measurement may uncover significant relationships not seen as a result of sole use of the %LR. The aLR was shown to increase as disease duration, levodopa duration, OFF motor scores, and LED increased. Moreover, an increase in aLR was significantly associated with increased levels of depression and freezing of gait, and a decline in cognition and perceived QOL. Thus, there may be a role for measuring the aLR on a yearly basis in patients at mid to late stages of PD to help plot disease trajectory. The amplitude of the aLR could help to indicate the onset of these other non-motor symptoms not indicated by the measure of %LR change. Utilization of the aLR in monitoring disease progression may have a direct impact on the decision-making process for interventions such as DBS in Parkinson disease.

## Ethics Statement

Seventy PD participants were recruited from the Movement Disorders Centre, University Hospital, London, Ontario, Canada. This study was approved by the Human Research Ethics Board (REB #107253) of Western University. All participants provided written informed consent prior to study enrollment and participation.

## Author Contributions

MP contributed to the design and execution of the study, analysis and interpretation of data, and production of the written article. SA contributed to the analysis and interpretation of data in the study. MJ contributed to the design of the study and revision of the written article.

## Conflict of Interest Statement

The authors declare that the research was conducted in the absence of any commercial or financial relationships that could be construed as a potential conflict of interest.
